# Dietary practices and nutritional status of young children in the former ensete monoculture dominated Sidama region, southern Ethiopia: A community based cross-sectional study

**DOI:** 10.1371/journal.pone.0272618

**Published:** 2022-09-14

**Authors:** Tsigereda Behailu, Selamawit Mengesha, Bernt Lindtjorn, Ingunn Marie S. Engebretsen

**Affiliations:** 1 College of Medicine and Health Sciences, Hawassa University, Hawassa, Ethiopia; 2 Centre for International Health, University of Bergen, Bergen, Norway; PLOS: Public Library of Science, UNITED KINGDOM

## Abstract

**Background:**

Child undernutrition is a challenge in Ethiopia, where morbidity and mortality among children are attributed to it. This study aimed to describe the dietary practices, household food insecurity, and nutritional status of young children in Dale district, Sidama region, southern Ethiopia. We discuss our findings in light of research from the same area 3 to 5 decades ago, and we analyze factors associated with linear growth of young children.

**Method:**

A community-based cross-sectional study design was employed. Children less than two years old and their primary caretakers (n = 903) were included in this study. Among whom 791 children were aged above six months and 742 children out of 791 provided a 24-hour dietary recall. Interviews capturing other dietary practices, food insecurity, socio-demographic characteristics, anthropometric measurements, and haemoglobin assessments were performed for all. The WHO Child Growth Standards were used to calculate anthropometric indices and to describe stunting (length-for-age z-score <-2). Haemoglobin measures below 11g/dl were defined as low haemoglobin levels (anaemia). Multilevel linear regression was used to identify factors associated with length-for-age z-scores.

**Result:**

The prevalence of stunting, wasting, and anaemia was 39.5%, 3.9%, and 61.7%, respectively. The majority of children (97%) ate cereals (maize) during the past 24 hours, and 79.6% of households use maize as the main food. Three fourth (75%) of the total households were food insecure ranging from mild to severe food insecurity. Boys were at higher risk of having lower length-for-age z-score than girls (β -0.53; 95% Confidence Interval (CI): -0.78, -0.27). For each month the children grew older, length-for-age z-score decreased slightly (β -0.06; -0.07, -0.04). Household food insecurity scores (β -0.05; -0.08, -0.01) and children haemoglobin levels, (β 0.21; 0.06, 0.35) were also associated with length-for-age z-score among young children.

**Conclusion:**

Linear growth failure (stunting) was prevalent among young children in the rural Sidama region and the majority of them were also anaemic. Older age, male sex, a lower haemoglobin level in children, and household food insecurity were risk factors of linear growth failure in young children. Maize seems to be the dominant food in this previously ensete dominated area; however, there have been minimal improvements in length-for-age z-score over decades. Strategies to ensure household’s food access and improve the quality of child diets need to be implemented.

## Introduction

Child undernutrition can be described both in relation to diet and nutritional status. Stunting, being too short for one’s age, is defined as a height/length-for-age Z-scores below minus two standard deviations (< -2 SD) from the Standard population mean [1] and height/lenth-for-age is an expression of attained linear growth performance. The global prevalence of child stunting in 2018 was 22% affecting over 149 million children under the age of five years [2].

In Ethiopia, the national prevalence of stunting among children under the age of five years was reported at 37% in 2019; which was 38% in 2016 [3, 4]. The national level prevalence of wasting was reported at 7% in 2019. In a similar national report, it was indicated that the prevalence of stunting and wasting in southern Ethiopia were 36.4% and 6.3, respectively in 2019, thus similar to the national average. A study done in Simada zone (currently Sidama region) in 2008 reported that 52% of children aged 12–23 months were stunted. Anaemia is also a major problem among children under the age of five years in Ethiopia, affecting 50% in 2016 [4]. A recent study from Wolayta zone, southern Ethiopia, also reported a high prevalence of anamia (65%) among children aged 6 to 23 months [5]. On top of this, only 11% of children aged 6–23 months in Ethiopia were getting a minimum acceptable diet in 2019 [3]. Sub-optimal child feeding practices are common in different parts of the country ranging from 42% in Addis Ababa to 98% in Somali [3]. The prevlaence of minimum dietary diversity was also reported at 35% in morth-west Ethiopia [6].

About 20 million people live in the southern and southwestern parts of Ethiopia where ensete (*Ensete ventricosum)* has been the major staple food [7]. The pseudostem and the corm of this “false banana” are used for food. However, the food contents show that the plant is rich in carbohydrates (80%), fibers (7%), and deficient in proteins (4%), fats (0.4%), vitamins, and other micronutrients [8, 9]. Most of the residents in rural Sidama are farmers who have been cultivating ensete as the main staple food for decades [8, 10, 11].

Ensete, also known as “the tree against hunger” has multiple benefits in addition to being a staple food and fodder for livestock, it is considered as being environment friendly [12, 13]. The people living in the ensete monoculture area were less affected by the drought and famine occurring in Ethiopia during the years from 1972 to 1985. Most recently other crops, particularly maize, tend to dominate due to the short harvesting time compared to ensete [14]. Cash crops such as fruits, coffee, and chat are covering large farms as income-generating resources, and ensete has gotten less attention. Moreover, a recent rapid population growth [15, 16] leading to increased population density has led to declining availability of farming areas. Therefore, the replacement of ensete with maize and other cash crops, and the rapid population growth, ultimately risk food insecurity. However, the impact of these changes on household food security and the nutritional status of children has not been adequately understood.

Children living in this ensete monoculture area have been affected by linear growth failure (stunting) for decades. Linear growth failure occurring during the first 1000 days of life is critical because of its negative consequences such as poor health, reduced learning capacity, and low economic productivity later in life [17–19]. However, few studies have reported on linear growth failure and its correlates among children less than two years old.

Our study aimed to describe nutritional status and dietary practices of young children less than two years, a critical period in which undernutrition develops, and to describe household food insecurity. In addition, we modelled how various factors affected linear growth. We are discussing our findings in light of high-quality nutritional studies done three to five decades ago. To our knowledge, limited nutritional studies have been done in this area in between those studies, and ours. Further, our study aimed to analyze the risk factors of linear growth faltering in the rural ensete monoculture dominated Dale district, Sidama region, southern Ethiopia.

## Materials and methods

### Study design and setting

A community-based cross-sectional study was conducted from August to November 2018 in seven randomly selected rural kebeles of Dale district. A kebele is the lowest administrative unit in the Ethiopian context. Dale district has a total of 36 rural and 2 urban kebeles. The main town in the district, Yirga Alem is located 320 km from Addis Ababa. The total population of Dale district was estimated at about 270,000 in 2017 and it is among the most densely populated districts (around 876 people/km^2^) in the country. The Dale district has 33 health posts, 10 health centres, and one referral hospital, Yirga Alem hospital. Most rural kebeles in the district have at least one health post operated by health extension workers. Services provided in the health posts are limited to immunization and family planning; no nutrition-based service was provided. Even though there is a non-governmental program, the productive safety net program (PSNP) working on severe household food insecurity, it is limited to a very few kebeles in the region [20].

Ensete (*Ensete ventricosum*) is widely cultivated in the area, and other food crops include maize, kale, cabbage, potato, and haricot beans. Coffee, chat (*Catha edulis*), and fruits like avocado and banana are the main cash crops grown in the area. The community also keeps livestock, and the livestock feed on ensete, and the animal manure is used as a natural fertilizer for the ensete cultivation.

### Study participants and sample size estimation

We visited homes with children younger than two years with mothers as the primary respondents or in her absence another primary caretaker. A baseline survey was conducted to list all eligible households in each of the study kebeles. Using a proportional allocation, households from each kebele were selected by a simple random method in statistical package for social science (SPSS) version 20 (IBM Corp, 2011). In households with more than one eligible child, one child was selected by a lottery method. If any of the children died or left the area before enrolment, the next household with a child under the age of two years was taken. A total of 91 replacements were made; two for the deaths of the selected children and the rest had permanently left the area.

The sample size was calculated using Open Epi version 3.01 (Dean AG, Sullivan Km, 2013) statistical software by assuming the proportion of the outcome variable; stunting, 95% confidence level, 4% precision, and 1.5 design effect. Although the Ethiopian demographic and health survey (2016) reported that the prevalence of stunting in southern Ethiopia was 39% [4], it was for children under the age of five years. Thus due to the intention to maximize our sample size, a proportion of 50% was assumed for this study. By adding a 10% non-response rate, the final sample size was set to 990 households with children under two years.

### Study variables

Dietary practices and nutritional status including length-for-age z-score (LAZ) and weight-for-length (WLZ) were described. Factors associated with LAZ were assessed including the child’s age, sex, birth order, and haemoglobin levels. Maternal characteristics included age, height stature, haemoglobin level, and educational status. Mother’s height was captured based on reported intergenerational effect [21] and previous studies in Ethiopia having identified it as important [22, 23]. Household factors included household’s food insecurity, wealth index, and family size.

### Data collection procedures

Data was collected through face-to-face interviews conducted in the local language, *Sidaamu Afoo*. A structured and pretested questionnaire was used to collect household socio-demographic and socio-economic characteristics, child background, feeding habits, and illness experiences. All the questionnaires used were prepared in English and translated to Amharic (the official language) and then to *Sidaamu Afoo* (the local language). Back translation was also done to check for consistency. Anthropometric measurements were done in the household’s compound and in places suitable to fix the measuring instruments; weight scales and length boards. Six data collectors (four nurses and two laboratory technicians) and two immediate supervisors were trained and participated in the data collection. The overall data collection activities were closely monitored by the investigators. Information on birth date and birth weight was obtained from the child immunization card. In the absence of an immunization cards, all the information was obtained from the respondent. In situations where the consented mother was not available for the interview for personal or practical reasons, mostly her consenting husband spoke on her behalf and provided some information related to her age and level of education. Measurements of her height, weight, and haemoglobin were taken during the interview visits or on other scheduled visits.

### Data quality control

Efforts such as confirmation of data with records like immunization cards and designing some questions in different ways for cross-checking were used. Calibration and regular cleaning of measuring instruments were done. Measurements (weight and length) were taken twice. Intensive training and close supervision of data collectors and using the local language for interviews were also additional strategies we pursued.

### Nutritional status of children

Digital SECA scales (SECA GmbH, Germany) were used to measure the weights of the child with minimal clothing. UNICEF length measuring boards with a fixed headboard, a movable footboard, and a ruler was used to take the recumbent length of the child to the nearest 0.1 cm. Length and weight were taken twice and recorded in a separate column as measurement one and measurement two. The average weight and length of children were calculated during data entry.

Anthropometric data and age in months were exported to Emergency Nutrition Assessment for Standardized Monitoring Assessment of Relief and Transitions software (Erhardt, Golden, and Seaman, 2011). Anthropometric indices were computed based on the World Health Organization, 2006 Child Growth Standards. The output was again exported to the statistical package for social sciences (SPSS) version 25 (IBM Corp, 2017) for further analysis. The prevalence of stunting and wasting were defined using WHO Child Growth Standards cut-off points [1], LAZ < -2, and WLZ < -2, respectively.

Haemoglobin levels of children and their biological mothers were measured with HemoCue HB 301 (Angelholm, Sweden) machines using finger-prick capillary blood samples. Haemoglobin levels were adjusted for altitude at 1500 metres and cut-off points were taken as 11g/dl for children [24] and 12.5g/dl for mothers [25] to determine the prevalence of anaemia. Illness experience was defined as the occurrence of any illness of the study child within two weeks preceding the survey.

### Dietary practices

Dietary diversity score was computed for children aged 6 to 24 months as that was the age range supposed to have started complementary feeding [26]. Data on the consumption of 10 food items by the child was obtained using a 24-hour dietary recall. The use of fats, sweets, and commercial/fortified foods by the child was also asked. The dietary diversity score was calculated from the 9 food items: cereals, roots and tubers, dairy products, legumes, flesh foods (including organ meats), eggs, fruits, green leafy vegetables, and other vegetables. The mean dietary diversity score and prevalence of minimum dietary diversity were computed based on the nine food groups. Minimum dietary diversity (MDD) was defined as the proportion of children aged between 6 and 24 months who received food from four or more food groups in the 24 hours prior to the study.

### Household food insecurity

Household Food Insecurity Access Scale (HFIAS) developed by the Food and Nutrition Technical Assistance (FANTA, 2007) and validated for use in Ethiopia [27] was employed to measure household food insecurity. The tool comprises nine questions 1) Uncertainty about food supply, 2) Unable to eat preferred food, 3) Eat a limited variety of food, 4. Eat foods really do not want to eat, 5) Eat smaller meals, 6) Eat fewer meals, 7) No food of any kind in the household, 8) Go to sleep at night hungry, and 9) Go a whole day and night without food. The tool measures the three food insecurity domains: uncertainty about food supply, poor dietary quality, and insufficient food intake and its physical consequences [27, 28]. Respondents were asked if they had faced any of those nine situations in the four weeks preceding this study. All ‘yes’ responses were followed by frequency questions coded with numbers; 1; if it happened once or twice, 2; if it happened three to ten times, and 3; if it happened more than ten times. All ‘No’ responses were scored ‘0’ for the frequency of occurrence. Households were classified into food secure, mildly food insecure, moderately food insecure, and severely food insecure based on Food and Nutrition Technical Assistance classification guidelines [29]. The HFIAS score ranged from 0 to 27, where a lower value was less and a higher value was more food insecurity. Categorical and continuous descriptive summaries are presented with tables and texts.

### Operational definition of household food insecurity categories

A food-secure household did not experience any food insecurity situation or only rarely experienced worry about food supply.

A mild food-insecure household is a household that experienced uncertainty about having enough food sometimes or often; and/or inability to eat the preferred food item rarely, sometimes or often; and/or eat a limited variety of food and eat a food that really do not want to eat rarely.

A moderate food-insecure household is a household that experienced eating a limited variety of food sometimes or often; and/or eating food that really did not want to eat sometimes or often; and/or eating smaller meals or fewer meals in a day rarely or sometimes.

A severe food-insecure household is a household that experienced eating smaller size of meals or fewer meals in a day often; and/or experienced any of the three most severe conditions of food insecurity; no food of any kind in the household, or going to bed hungry at night, or going a whole day and night without eating even if it occurred rarely.

### Wealth index

Wealth index was defined as a household’s economic status. Wealth index was computed by a principal component analysis (PCA) using possession of household assets such as radio, television, mobile phone, and livestock such as cow, sheep, goat, donkey, and hens [30, 31]. The Kaiser Meyer Olkin (KMO) sampling adequacy test was 0.62 with a significant level (<0.001) Bartlett’s Test of Sphericity. Four out of nine components had an eigenvalue greater than 1. Finally, households were ranked based on percentile scores and grouped into three economic statuses: very poor, poor, and least poor.

### Data cleaning

**Fig 1:**
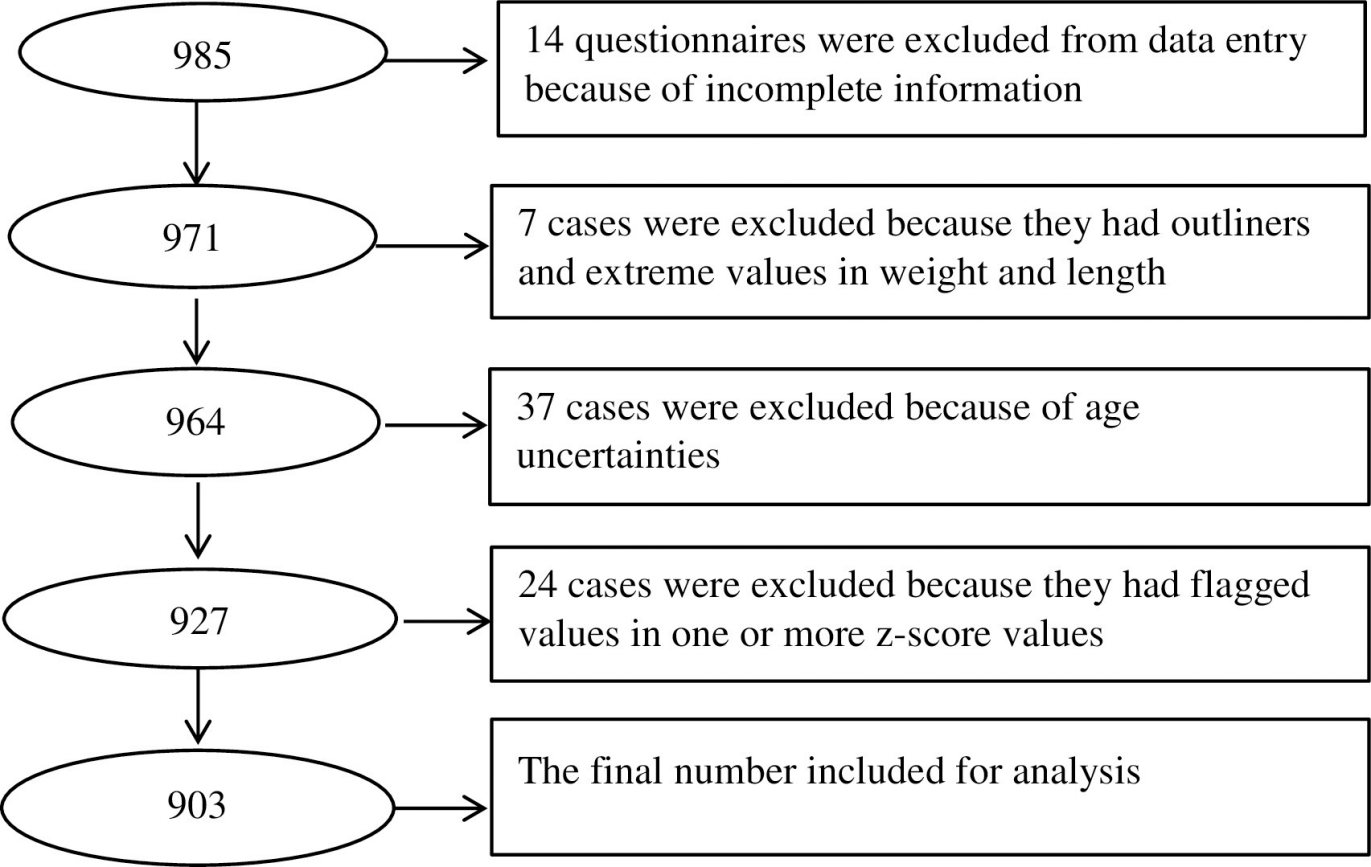
Flow chart showing a stepwise exclusion of the study subjects during data cleaning and analysis.

Data cleaning started at the time of data collection. All questionnaires were checked for completeness and any missing information was recaptured. The data was double entered and validated using Epi Data 3.1 (Epi Data Association, Odense, Denmark) software. SPSS version 25 (IBM Corp, 2017) was used to check for any missing values, outliers, and inconsistent information. A total of 82 cases were excluded from the analysis due to multiple missing values which were difficult to recapture, the uncertainty of child’s age, and other reasons as shown with Fig 1. Visit dates and birth dates were converted to the Gregorian calendar from the Ethiopian calendar using date conversion application software.

### Statistical analysis

Data analysis was done using SPSS version 25 (IBM Corp, 2017), and STATA version 12 for windows (Stata Corp LP, College Station, TX, 2011). Descriptive statistics; means, medians, frequencies, and percentages are used to present the continuous and categorical variables. Bivariate linear regression was carried out for 20 independent variables to identify variables with a p-value less than 0.25 at a 95% CI for multivariable linear regression. Multicollinearity was also checked for the selected independent variables using collinearity diagnostics. Crude and adjusted linear regression models were applied to describe associations with anthropometric indices, length-for-age z-score (LAZ). Statistical significance was set at p-value ≤ 0.05 and the regression coefficients with the 95% confidence interval (CI) were reported. To account for clustering at kebele level, (ICC = 0.051), adjustment with the vce option in STATA was used for our final model. The model performance was assessed with the model goodness of fit which was not significant.

### Ethical considerations

Ethical clearance was obtained from Hawassa University, Ethiopia (Ref. No IRB/025/10, Date: 21/12/2017) and the Ethical committee of Western Norway, Norway (Ref: 2017/90/REK, Date: 07/03/18). All the necessary official letters were obtained from the concerned bodies. Written informed consent was obtained from respondents and legal guardians of children, usually the mother or the father. In the case of illiterate respondents, the data collector had to read the subject information and obtained a finger stamp when they agreed to participate. The data is kept in a secure place to maintain confidentiality. Furthermore, identifiers like household identification numbers were not used to communicate findings. This study was carried our following all applicable standards principles of the World Medical Association Declaration of Helsinki, 2013 [32].

## Result

### Background information

Out of the 985 children whose guardians consented to data collection, 903 (91.7%) were analysed in this paper (Fig 1). Of the 903 respondents, 876 (97%) were mothers, and the rest 27 (3%) were non-maternal caregivers, of whom 2 mothers had died and no maternal data was obtained. Non-maternal caregivers, mostly fathers, provided information about the mother (n = 21) or stepmother (n = 4). We had measurement data from the 876 responding and the 21 non-responding biological mothers, in total 897. The mean (SD) household size was 4.8 (1.6) and 459 (50.8%) of the 903 households had 2 to 4 members (Table 1). In 808 (89.5%) of the total households, there was only one child who was under the age of five years (including the index child). Maize was the main food for 719 (79.6%) of the total households and only 175 (19.4%) of them reported using ensete as the main food. Majority, 713 (79%) of the total households, used their production as the main source of food. For 362(40.1%) of the total households, a protected well or spring was the main source of water (Table 1). Near to three-fourth, 661 (73.2%) of the total households were keeping cows and more than half, 529 (58.6%) of the 903 households owned hens.

**Table 1:** Household factors and characteristics of mothers of the study children in Dale district, Ethiopia, 2018 (n = 903, except where otherwise indicated).

Variable	Category	Frequency (N)	Percent (%)
Respondent	Mother	876	97.0
	Father	16	1.8
	Others	11	1.2
Family size	2–4	459	50.8
	5–7	387	42.9
	>7	57	6.3
Children < 5 years of age in the HH	One	808	89.5
	More than one	95	10.5
Wealth status	Very poor	279	30.9
	Poor	325	36.0
	Least poor	299	33.1
Common food used for the family	Maize	719	79.6
	Ensete	175	19.4
	Teff and other	9	1.0
Main source of food for the HH	Household farm	713	80.0
	Local marketing	187	20.7
	Other sources	3	0.3
Main source of water for the HH	Tap-water	350	38.8
	Protected well/spring	362	40.1
	Unprotected well/spring	164	18.2
	Other sources	27	3.0
Age of the mother (n = 897)	15–24 years	282	31.4
	25–34 years	518	57.7
	35–49 years	97	10.8
Mother’s educational status (n = 897)	No education	160	17.8
	Primary school	407	45.3
	Secondary school	330	36.8
Total number of pregnancies (n = 897)	One	260	29.0
	Two to three	376	41.9
	Four and above	261	29.1
Age at first pregnancy (n = 897)	< 20 years	791	88.2
	20 to 35 years	91	10.1
	Do not remember	15	1.7
Hemoglobin level of the mother (897)	< 12.5 g/dl	158	17.6
	**≥ 12 g/dl**	**739**	**82.4**

CI = confidence interval.

HH = household.

### Maternal characteristics

The age range of mothers (n = 897) was 16–45 years and the mean (SD) age was 26.9 (5.1) years. More than 40% (407 of the 897) had attended primary school education and 5 (0.5%) of the 897 mothers were employed. The mean (SD) age of mothers at first pregnancy was 17.9 (1.8) years while 15 mothers did not remember their age at first pregnancy. About 30% (264) of the 897 mothers had only one pregnancy. Haemoglobin levels were recorded less than 12.5g/dl for 158 (17.6%) of the 897 mothers (Table 1).

### Child characteristics

Of the 903 children (447 male and 456 female) who participated in our study, 537 (59.5%) were aged 12–24 months (Table 2). The mean (SD) age was 14.1 (6.3) months (S1 Table). The immunization card was accessed for 547(60.6%) of 903 children. More than half of the total children were born at health centres 430 (47.6%) or hospitals 120 (13.3%). Of the 903 children, 265 (29.3%) were recorded as the first child to their mother. Information on birth weight was obtained for 722 (80%) of the total children and 249 (34.5%) of them were confirmed from the immunization cards. Illness during two weeks prior to this study was reported by 72 (8%) of 903 children; majority of them (40 of 72) experienced cough or cough and fever (Table 2).

**Table 2:** Child characteristics and young child feeding practices in Dale district, Ethiopia, 2018, categorical variables (n = 903, except where otherwise indicated).

Variable	Category	Frequency (N)	Percent (%)
Age in months	< 6 month	112	12.4
	6–12 month	254	28.1
	12–24 months	537	59.5
Sex of the child	Male	447	49.5
	Female	456	50.5
Birth order	First child	265	29.3
	Second and above	638	70.7
Place of birth	Home	344	38.1
	Health center	430	47.6
	Hospital	120	13.3
	Other	9	1.0
Birth weight (N = 722)	< 2.5 kg	93	12.9
	≥ 2.5 kg	629	87.1
Breastfeeding initiation	Within the 1st hour after birth	698	77.4
	After the 1^st^ one hour after birth	204	22.6
Exclusive Breastfeeding (Children < 6 months, n = 112)	Yes	88	78.6
	No	24	21.4
Complementary Feeding (6–24 months age, n = 791)	Yes	742	93.8
	No	49	6.2
Illness in the past two weeks	Yes	72	8.0
	No	831	92.0
Type of illness (N = 72)	Cough	31	43.1
	Diarrhea	7	9.7
	Fever	8	11.1
	Cough and fever	9	12.5
	Diarrhea and fever	15	20.8
	Other	2	2.8

### Nutritional status of children aged 0 to 24 months

The prevalence of stunting (LAZ < -2) was 39.5% (357out of 903 children) and out of them 163 (45.7%) were severely stunted (LAZ < -3). The mean (SD) length-for-age z-score was -1.6 (1.7). The prevalence of wasting was 3.9% (35 out of 903) and out of them, 12 (34.3%) were severely wasted (WLZ < -3). The adjusted haemoglobin level of 557 (61.7%) of the 903 children was below 11g/dl (Table 2). The descriptive output for haemoglobin level, weight, length, anthropometric indices, and other continuous variables are presented in S1 Table.

### Dietary practices

Almost all, 896 (99.2%) of the total children were being breastfed at the time of data collection. Among the 7 children not being breastfed, all were above 6 months; two had lost their mothers, and 5 had different reasons like separation from the mother and occurrence of new pregnancy. More than three-fourth (698 of 903) the mother-child dyads had started breastfeeding with in the first one hour after delivery.

Although all children below the age of six months (112) were breastfed, exclusive breastfeeding was practiced by 88(78.6%) of 112 children aged below six months (Table 2). The rest 24/(21.4%) of them had started taking food. Out of 791 children aged 6 to 24 months, 49 (6.2%) children had not started complementary feeding. Out of them, 25 were aged 6 to 7 months, 20 were aged 7 to 8 months, and 3 were aged 8 to 9 months, and one child was above 9 month. Out of the 742 children aged above six months who had started complementary feeding, 16 (2.0%) reported that they had started complementary feeding before the age of six months (Table 2).

Dietary diversity score was computed for children above six months who had started complementary feeding, 742 (82.2%) of 903 children. The mean (SD) dietary diversity score was 4.3 (1.9) and the minimum score was 1 out of 9 and the maximum score was 9 out of 9. Minimum dietary diversity, defined as consumption of foods from five or more food groups, was practiced by 452 (39.1%) of 742 children. The common food group consumed were cereals 720 (97%) followed by dairy products, 557 (75.1%) of the 742 children. More information can be seen in Table 3.

**Table 3:** Consumption of food groups during the past 24 hours in rural Dale district, Ethiopia, 2018 (n = 742).

	Consumption in the past 24 hours			
	Yes		No	
List of food groups	Frequency (N)	Percent (%)	Frequency (N)	Percent (%)
Cereals, including maize	720	97.0	22	3.0
Roots and tubers including ensete	547	73.7	195	26.3
Legumes and nuts	96	12.9	646	87.1
Dairy products	557	75.1	185	24.9
Flesh foods	24	7.3	688	92.7
Eggs	272	36.7	470	63.3
Green leafy vegetables	298	40.2	444	59.8
Other vegetables	141	19.0	601	81.0
Fruits of all types	476	64.2	266	35.8
Fats and oils	411	55.4	331	44.6
Sugar and sweets	147	19.8	595	80.2
Commercial/fortified foods	75	10.1	667	89.9

### Household food insecurity status

The mean (SD) household food insecurity score was 4.0 (3.2). Three-fourth (677) of the total households were food insecure and prevalence of severe food insecurity was 17.8% (161 of 903 households). The highest affirmative answer, 556 (61.6) of 903 was given for ‘Eating a limited variety of foods’ followed by ‘worry about having enough, 517 (61.1%) of 903. The occurrence frequencies for the nine household food insecurity conditions are presented in Table 4.

**Table 4:** Households’ experience and frequency of occurrence of food insecurity conditions in Dale district, Ethiopia, 2018, during the past four weeks (n = 903).

	Frequency of occurrence over the past four weeks			
Household food insecurity conditions	Never	Rarely	Sometimes	Often
	Frequency (%)	Frequency (%)	Frequency (%)	Frequency (%)
Worried about getting enough food	386 (42.7)	348 (38.5)	157 (17.4)	12 (1.3)
Unable to eat preferred foods	390 (43.2)	317 (35.1)	190 (21.0)	6 (0.7)
Ate a limited variety of foods	347 (38.4)	310 (34.3)	181 (20.0)	65(7.2)
Ate foods that really did not want to eat	715(79.2)	142 (15.7)	42 (4.7)	4 (0.4)
Ate a smaller meal	571 (63.2)	206 (22.8)	122 (13.5)	4 (0.4)
Ate fewer meals in a day	640 (70.9)	186 (20.6)	74 (8.2)	3 (0.3)
No food of any kind in the household	814 (90.1)	59 (6.5)	29 (3.2)	1 (0.1)
Went to sleep at night hungry	805 (89.1)	79 (8.7)	19 (2.1)	0
Went a whole day and night without eating	860 (95.2)	35 (3.9)	7 (0.8)	1 (0.1)

### Association of independent variables with length-for-age Z score

The result of the bivariable and multivariable linear regression models are presented in Table 5. The age, sex, haemoglobin level of the child and household food insecurity score were independently associated with length-for-age z-score (LAZ). LAZ decreased by 0.06 (β-0.06; -0.07, -0.04) when the age increased by one month. Male sex of the child was associated with a decrease of LAZ by 0.53 (β-0.53; -0.78, -0.27) compared to being female. An increase in the haemoglobin level of the child by 1g/dl was associated with an increase in LAZ by 0.21 (β 0.2; 0.06, 0.35). When the household food insecurity score increased by one (more food insecure), LAZ decrease by 0.05 (β-0.05; -0.09, -0.01).

**Table 5:** Effect of selected predictor variables on length-for-age z-scores among 903 children 0 to 24 months of age in Dale district, southern Ethiopia, 2018.

Variables	Unadjusted coefficients	95% CI for the unadjusted coefficients	Adjusted coefficients	95% CI for the adjusted coefficients
Age in months	**-0.05**	**-0.07, -0.04**	**-0.06**	**-0.07, -0.04**
Male sex	**-0.56**	**-0.91, -0.21**	**-0.53**	**-0.78, -0.27**
Birth order	**0 .12**	**0.05, 0.20**	0.07	-0.1, 0.3
Child hemoglobin	**0.18**	**0.02, 0.34**	**0.21**	**0.06, 0.35**
Mother’s age	0 .02	-0.01, 0.04	-0.01	-0.05, 0.03
Mother’s height	0.02	-0.01, 0.04	0.03	-0.01, 0.06
Education of mother				
Primary school education^1^	-0.39	-0.84, 0.05	-0.33	-0.87, 0.23
Secondary school education^1^	**-0.41**	**-0.79, -0.03**	-0.26	-0.75, 0.22
HFI score	-0.05	-0.12, 0.02	**-0.05**	**-0.09, -0.01**
Family size	0.11	0.02, 0.2	0.04	-0.1, 0.18
Wealth status				
Poor^2^	0.05	-0.44, 0.53	0.09	-0.31, 0.49
Least poor^2^	-0.26	-0.97, 0.44	-0.24	-0.86, 0.39

^1^Compared with no education (the reference category).

^2^Compared with very poor (the reference category).

CI = confidence interval.

## Discussion

Our finding showed that linear growth failure among rural young children was high 39.5%. About 60% of young children were not getting the minimum dietary diversity and 75% of the households were food insecure. The majority were initiating breastfeeding within one hour and exclusive breastfeeding was practiced to a high degree. Although complementary feeding had started at the right time for most babies, it was also delayed for 6% of them. Thus, food insecurity, incomplete diet and lack of complementary feeding were nutritional threats among the young children in this study. The majority of the households used maize as a staple food. Older child age, being male, lower haemoglobin levels of the child, and household food insecurity were associated with lower LAZ scores.

We discuss our findings in light of studies done 3 and 5 decades ago and focus on the areas of infant and toddler dietary practices and child anthropometry. Risk factors for linear growth failure are discussed in light of current literature. One of the oldest studies was conducted in 1971 [10] and of relevance to us is their presentation of the child diet. The other study was done in 1993 [33] and it presents child anthropometry.

### Child nutritional status

The study from the 90-ties evaluated the nutritional impact of seasonality in children [33]. The study documented that the mean height-for-age z-score was -2.2, which is lower than the mean (SD) in our study, -1.6 (1.7). Although we used WHO (2006) Growth Standards as a reference, F. Branca and colleagues used WHO reference values from 1983 (NCHS reference population), there are reasons to believe that for longitudinal growth impairment, the new standard corresponds to slightly higher values than in the old reference. Therefore, the mean difference would be the difference between -2.2 and -1.6 or more, not less. One can expect an improvement of one-half of a standard deviation in longitudinal growth over 30 years which is seemingly a small improvement over such a long period with a high level of undernutrition. The prevalence of stunting (39.5%) is still alarming.

### Dietary practices

Our study demonstrated a change in the staple food of the rural community of Sidama region compared to studies carried out decades ago. In our study, maize was the commonly used staple food for about 80% of the households, and ensete was found less prominent in the diet; nearly 20%. In the 1970-ties [10] it was reported that ensete was the main staple food of the family including young children. However, in our study ensete was the second common food used for young children next to maize. Thus, there is a reason to believe there has been a dietary shift from ensete to maize among the rural residents of the region. This dietary shift might be due to the adaptation of the newly introduced crop, maize, which is considered as the modern approach [14]. Moreover, the cultivation of crops like maize, coffee, chat, and fruits might be an income-generating activity. Crop diversification is encouraged to improve dietary diversity [34] and sustainability. On the other hand, draught and poverty, may lead to unsustainable choices or risk taking and ultimately increased vulnerability. As a result there may be a progressive decline in ensete production [14]. In the 70-ties it is described that kale was always served with ensete. However, in our study, green leafy vegetables were served to 40% of children during the past 24 hours. These may indicate that vegetable consumption has gone down and that toddlers are eating less nutritious vegetables than recommended.

The previous study [10] reported that it was a culture feeding fresh butter, boiled water, and fenugreek home preparations from the day of birth. Such cultural practices play an important role in disturbing the normal digestion among young children, hence causing undernutrition and ill health. However, in our study, pre-lacteal foods were given to 4.3% of the children, and exclusive breastfeeding was practiced among 78% of children under the age of six months. This may indicate emphasized messages that changed practices in favour of breastfeeding. In our study, bottle-feeding was used for 2.2% of the children, which was not practiced in the previous studies. Bottle-feeding should not be a primary choice in situations where mothers can spend adequate time with their babies. Even though the use of bottle-feeding seems to be low (2.2%), it is considerable for a rural community where less than 1% of mothers were employed.

Milk was consumed by 75% of the children in our study during the past 24 hours. It was served diluted with water or after traditional extraction of the fat (butter). This may be due to prior public health messages on what to do when breastfeeding cannot be achieved and formula milk is unaffordable [35, 36]. The current use of diluted cow’s milk where breastfeeding is almost universal needs further explanation and understanding. Reasons for using diluted milk may include separation from the mother and the need for alternative feeds. The need for earning money from selling the milk or fat (butter) extracted might give an additional explanation [37]. Since milk is the only animal source food given to children in the study area, critical consideration of its quality is needed. It may be possible to conclude that children nowadays get a diet less calorie-dense than earlier as fats and oils only were served to half of the children above 6 months.

In the 70-ties [10] animal-source protein intake was reported low similar to our study where only 7.3% got flesh foods. In our study, legumes and nuts were served for only 13% of the children. Therefore, it is a potential to improve protein-rich food intake using both animal and plant sources. However, there were food taboos described in the 70-ties, which could not be confirmed in our study, thus, there may be potential for recommendation of more nutritious complementary diets

### Risk factors of linear growth faltering (stunting)

The prevalence of stunting in our study was similar to EDHS (2016) from the Southern region [4]. However, our study subjects were children under the age of two years from a typical rural area; the EDHS report had included children under the age of five years from both urban and rural areas. This indicates that stunting persists from the early ages. EDHS (2016) reported that stunting had its peak at 24 months; similarly, our finding supports that older children were more affected by stunting. This finding has its implication for indicating that the critical period for prevention of stunting is before the age of two years.

Being male was associated with a decrease in the length-for-age z-score almost by half which is consistent with the EDHS (2016) report and other studies [22, 38–41]. A study done in four regions of Ethiopia support our finding; stunting is common among male children than girls [42] However, there are studies reporting no association between stunting and child’s sex [43–45], and that boys are at a lower risk than girls [46]. The observed sex differences have not yet been fully explained, neither with biological, dietary, or cultural differences. One suggested explanation from a study in Senegal was that boys end exclusive breastfeeding earlier than girls in the recommended exclusive breastfeeding period of the first six months [47]. Our study did not collect data to assess this and we suggest further study to deeply investigate the nutritional status in relation to gender differences.

Similarly, haemoglobin level of the child was positively associated with the linear growth of a child. We have found that each unit of haemoglobin level was associated with an increase of LAZ score by 0.21. Besides, 27% of the children aged 0–24 months were anaemic and stunted. This relationship has recently been described by other child health studies from southern Ethiopia [48, 49], which suggested looking at anaemia and stunting as syndemic needing similar preventive strategies. Another study done in India and Peru support our finding; positive association between linear growth failure and low haemoglobin level [50].

In our study, only 25% of the households were food secure. Even though the older studies from the study area did not report on household food insecurity, recent studies from the region agree with our findings [51, 52]. In our study, an increase in the household food insecurity score was associated with a decrease in LAZ scores. This finding is consistent with a recent study done in other regions of Ethiopia [53]. Actions are needed to improve the situation where only a quarter feel food secure. Maternal factors; height and haemoglobin did not demonstrate association in our study, however maternal height was a risk factor in previous studies [54].

### Strengths of the study

Our study was a community-based study, with random selection of households, reaching less accessible rural areas. The sampling frame was prepared by a baseline survey conducted prior to the main data collection. Comprehensive data were collected at an individual level (the young child and the mother) and the household level. All the data collectors and supervisors were fluent speakers of the local language, *Sidaamu Afoo* which was used for the interview. Simulated practical training was included in the training session of data collectors and supervisors.

### Limitations of the study

Our study may be subject to information bias; self-reported data like age, birth weight, breastfeeding initiation time, and dietary recalls may create informational bias. Measurement bias is also expected to exist. Replacement of some households who left the area before enrolment may create a selection bias. Almost 8% of the visited households were excluded from data analysis due to child age uncertainty and incomplete key data necessary for our analysis. This may be considered relatively high but indicates challenges in capturing such type of data in a rural setting with low level of education, a different calendar system, and lack of health records. However, we performed the sample size calculation by considering a 10% non-response rate. Also, there may be residual confounding not addressed particularly including long term morbidity patterns such as parasites and immune deficiency.

## Conclusion

This study demonstrated that there is high burden of chronic child undernutrition. According to older studies from the area, there have been limited improvements over decades. It also identified changes in the food system of the community where the cultivation and utilization of ensete is being replaced by maize. Older child age, male sex, lower haemoglobin levels of the child, and household food insecurity were associated with lower linear growth. There is a need to strengthen efforts to improve household food security, young children’s complementary diet and economic status with considerable focus in rural districts while monitoring growth, diets, and health in young children. Furthermore, promoting the environmental benefit of ensete and availing modern technologies for ensete cultivation and processing can be a strategic approach.

## Supporting information

S1 Table Descriptive findings of continuous variables among children and their mothers in Dale district, Ethiopia, 2018. (PDF)

S1 File Raw data used to constract Tables 1–5 in Dale district, Ethiopia, 2018. (SAV)

S2 File Appendix (Questionnaire; English version). (PDF)

S3 File Appendix (Questionnaire; Sidaamu Afoo version). (PDF)

## References

[1] World Health Organization (2006) Child Growth Standards based on length/height, weight and age, in *Acta paediatrica (Oslo, Norway: 1992) Supplement*. pp. 76. 10.1111/j.1651-2227.2006.tb02378.x 16817681

[2] Food and Agriculture Organization (2014) FAO. *The State of Food Insecurity in the World*. 2014. *www.fao.org/3/a-i4030e.pdf*.

[3] *Ethiopian Public Health Institure and ICF (2019) Ethiopia mini demographic and health survey 2019: key indicators*. Rockville, Maryland, USA: EPHI and ICF.

[4] Central Statstical Agency and ICF (2016) Ethiopia Demographic and Health Survey 2016: Key Indicators Report. *International Journal for Equity in Health*. p. 32–45.26912255

[5] Alemayehu M, Meskele M, Alemayehu B (2019) Prevalence and correlates of anemia among children aged 6–23 months in Wolaita Zone, *Southern Ethiopia*. 14: e0206268. 10.1371/journal.pone.0206268. PMC6407854 30849088

[6] Abebe Z, Tariku A, Bikes GA, Wassie MM, Gonete KA, et al. (2019) Poor child complementary Feeding Practices in northwest Ethiopia: Finding from the Baseline Survey of Nutrition Project, 2016. Ital J Pediatr45: 154. 10.1186/s13052-019-0747-2. PMC6889572 31791372

[7] Yemata G (2020) Ensete ventricosum: A Multipurpose Crop against Hunger in Ethiopia. ScientificWorldJournal, 2020: 6431849. 10.1155/2020/6431849. PMC7199586 32395087

[8] Mohammed B, Gabel M, Karlsson LM (2013) Nutritive values of the drought tolerant food and fodder crop enset. African Journal of Agricultural Research8: 2326–2333. 10.5897/AJAR12.1296.

[9] Nurfeta A, Tolera A, Eik LO, Sundstøl F (2008) Yield and mineral content of ten enset (Ensete ventricosum) varieties. Trop Anim Health Prod 40: 299–309. doi: 10.1007/s11250-007-9095-0 18557193

[10] Selinus R, Gobezie A, Vahlquist B (1971) Dietary studies in Ethiopia. 3. Dietary pattern among the Sidamo ethnic group. A study on villagers in the enset monoculture area in S. *Ethiopia with special attention to the situation in young children*. Acta Societatis Botanicorum Poloniae76: 158–178. 5135499

[11] Sonko A (2016) Assessment of dietary practice and anthropometric status of pregnant women in Aleta Chuko Woreda Southern Nations, Nationalities and People’s Region/SNNPR/, Ethiopia. J Epidemiol Public Health Rev 1: 1–9. 10.16966/2471-8211.102.

[12] Borrell JS, Biswas MK, Goodwin M, Blomme G, Schwarzacher T, et al. (2019) Enset in Ethiopia: a poorly characterized but resilient starch staple. Ann Bot 123: 747–766. 10.1093/aob/mcy214. 30715125 PMC6526316

[13] Borrell JS, Goodwin M, Blomme G, Jacobsen K, Wendawek AM, et al. (2020) Enset‐based agricultural systems in Ethiopia: A systematic review of production trends, agronomy, processing and the wider food security applications of a neglected banana relative. Plants, People, Planet2: 212–228. 10.1002/ppp3.10084.

[14] Quinlan RJ, Quinlan MB, Dira S, Caudell M, Sooge A, et al. (2015), Vulnerability and resilience of Sidama enset and maize farms in Southwestern Ethiopia. Journal of Ethnobiology35: 314–336. 10.2993/etbi-35-02-314-336.1.

[15] Quinlan RJ, Dira SJ, Caudell M, Quinlan MB (2016) Culture and psychological responses to environmental shocks: Cultural ecology of Sidama impulsivity and niche construction in southwest Ethiopia. Current Anthropology. 10.1086/688213.

[16] Areru HA, Dangisso MH, Lindtjørn B (2020) *Births and deaths in Sidama in southern Ethiopia: findings from the 2018 Dale-Wonsho Health and Demographic Surveillance System (HDSS)*. 13: 1833511. 10.1080/16549716.2020.1833511. PMC7598947 33115376

[17] Oot L, Sethuraman K, Ross J, Sommerfelt A (2016) The Effect of Chronic Malnutrition (Stunting) on Learning Ability, a Measure of Human Capital: A Model in PROFILES for Country-Level-Advocacy. Food and Nutrition technical Assistance3: 1–8.

[18] Woldehanna T, Behrman JR, Araya MW (2017) The effect of early childhood stunting on children’s cognitive achievements: Evidence from young lives Ethiopia. Ethiopian Journal of Health Development31: 75–84. 29249889 PMC5726774

[19] Wolde T, Belachew T (2019) Chronic undernutrition (stunting) is detrimental to academic performance among primary schools of adolescent children: a randomized cross sectional survey in Southern Ethiopia. BMC Res Notes12: 142. doi: 10.1186/s13104-019-4160-0 30876451 PMC6419846

[20] Galato ZG (2020) Impacts of Productive Safe Southern Ethiopia. International Journal of Current Research Key Words.

[21] Martorell R, Zongrone A (2012) Intergenerational influences on child growth and undernutrition. Paediatr Perinat Epidemiol 26 Suppl 1: 302–14. 10.1111/j.1365-3016.2012.01298.x. 22742617

[22] Medhin G, Hanlon C, Dewey M, Alem A, Tesfaye F, et al. (2010) Prevalence and predictors of undernutrition among infants aged six and twelve months in Butajira, Ethiopia: the P-MaMiE Birth Cohort. BMC Public Health10: 27. doi: 10.1186/1471-2458-10-27 20089144 PMC2826285

[23] Dessie ZB, Fentie M, Abebe Z, Ayele TA, Muchie KF (2019) Maternal characteristics and nutritional status among 6–59 months of children in Ethiopia: further analysis of demographic and health survey. BMC pediatrics19: 1–10. doi: 10.1186/s12887-019-1459-x 30894145 PMC6425564

[24] Sullivan KM, Mei Z, Grummer-Strawn L, Parvanta I (2008) Haemoglobin adjustments to define anaemia. Tropical Medicine & International Health13: 1267–1271. 10.1111/j.1365-3156.2008.02143.x. 18721184

[25] Neufeld L, García-Guerra A, Sánchez-Francia D, Newton-Sánchez O, Ramírez-Villalobos MD, et al. (2002) Hemoglobin measured by Hemocue and a reference method in venous and capillary blood: a validation study. Salud pública de México44: 219–227. doi: 10.1590/s0036-36342002000300005 12132319

[26] Sinhababu A, Mukhopadhyay DK, Panja TK, Saren AB, Mandal NK, et al. (2010) Infant-and young child-feeding practices in Bankura district, West Bengal, India. Journal of health, population, and nutrition28: 294. doi: 10.3329/jhpn.v28i3.5559 20635641 PMC2980895

[27] Gebreyesus SH, Lunde T, Mariam DH, Woldehanna T, Lindtjørn B (2015) Is the adapted Household Food Insecurity Access Scale (HFIAS) developed internationally to measure food insecurity valid in urban and rural households of Ethiopia? BMC Nutrition1: 2.

[28] Swindale A, Bilinsky P (2006) Development of a universally applicable household food insecurity measurement tool: process, current status, and outstanding issues. The Journal of nutrition136: 1449S–1452S. 10.1093/jn/136.5.1449S. 16614442

[29] Coates J, Swindale A, Bilinsky P (2007) *Household Food Insecurity Access Scale (HFIAS) for measurement of food access: indicator guide: version 3*.

[30] Pirani E(2014) Wealth index. Encyclopedia of Quality of Life and Well-Being Research. Springer, Dordrecht. 10.1007/978-94-007-0753-5_3202.

[31] Vyas S, Kumaranayake L (2006) Constructing socio-economic status indices: how to use principal components analysis. Health Policy and Planning21: 459–468. doi: 10.1093/heapol/czl029 17030551

[32] Helsinki WMADo (2013) World Medical Association Declaration of Helsinki: ethical principles for medical research involving human subjects. Jama310: 2191–4. 10.1001/jama.2013.281053 24141714

[33] Branca F, Pastore G, Demissie T, Ferro-Luzzi A (1993) The nutritional impact of seasonality in children and adults of rural Ethiopia. European journal of clinical nutrition47: 840–850. 8156981

[34] Mellisse BT, van de Ven GW, Giller KE, Descheemaeker K (2018) Home garden system dynamics in Southern Ethiopia. Agroforestry Systems 92: 1579–1595. 10.1007/s10457-017-0106-5.

[35] World Health Organization (2005) *Guiding principles for feeding non-breastfed children 6–24 months of age*: World Health Organization.

[36] Riley LK, Rupert J, Boucher O (2018) Nutrition in Toddlers. Am Fam Physician98: 227–233. 30215978

[37] Tolosa T, Verbeke J, Piepers S, Tefera M, Getachew Y, et al. (2016) Milk production, quality, and consumption in Jimma (Ethiopia): Facts and producers’, retailers’, and consumers’ perspectives. Prev Vet Med124: 9–14. 10.1016/j.prevetmed.2015.12.016. 26763115

[38] Tafesse T, Yoseph A, Mayiso K, Gari T (2021) Factors associated with stunting among children aged 6–59 months in Bensa District, Sidama Region, South Ethiopia: unmatched case-control study. BMC pediatrics21: 1–11. doi: 10.1186/s12887-021-03029-9 34872503 PMC8647487

[39] Wamani H, Astrøm AN, Peterson S, Tumwine JK, Tylleskär T (2007) Boys are more stunted than girls in sub-Saharan Africa: a meta-analysis of 16 demographic and health surveys. BMC Pediatr7: 17. doi: 10.1186/1471-2431-7-17 17425787 PMC1865375

[40] Teshome B, Kogi-Makau W, Getahun Z, Taye G (2009) Magnitude and determinants of stunting in children underfive years of age in food surplus region of Ethiopia: the case of west gojam zone. Ethiopian Journal of Health Development23. 10.4314/ejhd.v23i2.53223.

[41] Jiang Y, Su X, Wang C, Zhang L, Zhang X, et al. (2015) Prevalence and risk factors for stunting and severe stunting among children under three years old in mid‐western rural areas of C hina. Child: care, health and development41: 45–51. 10.1111/cch.12148. 24797895

[42] Hirvonen K, Wolle A, Laillou A, Vinci V, Chitekwe S, et al. (2021) Child growth faltering dynamics in food insecure districts in rural Ethiopia. Maternal & child nutrition e13262. doi: 10.1111/mcn.13262 34523809 PMC11258764

[43] Woldie YT, Belachew T, Hailu D, Teshome T, Gutema H (2015) Prevalence of stunting and associated factors among under five children in Wondo Genet Woreda, Sidama Zone, Southern Ethiopia. International Journal of Medical and Health Sciences Research2: 36–49. 10.18488/journal.9/2015.2.2/9.2.36.49.

[44] Gari T, Loha E, Deressa W, Solomon T, Lindtjørn B (2018) Malaria increased the risk of stunting and wasting among young children in Ethiopia: Results of a cohort study. PLoS One 13: e0190983. doi: 10.1371/journal.pone.0190983 29324840 PMC5764317

[45] Belayneh M, Loha E, Lindtjørn B (2020) Seasonal Variation of Household Food Insecurity and Household Dietary Diversity on Wasting and Stunting among Young Children in A Drought Prone Area in South Ethiopia: *A Cohort Study* 1–26. 10.1080/03670244.2020.1789865. 32672490

[46] Abeway S, Gebremichael B, Murugan R, Assefa M, Adinew YM (2018) Stunting and its determinants among children aged 6–59 months in northern Ethiopia: a cross-sectional study. Journal of nutrition and metabolism 2018. 10.1155/2018/1078480. PMC6036796 30046469

[47] Bork KA, Diallo A (2017) Boys are more stunted than girls from early infancy to 3 years of age in rural Senegal. The Journal of nutrition147: 940–947. doi: 10.3945/jn.116.243246 28298540

[48] Orsango AZ, Habtu W, Lejisa T, Loha E, Lindtjørn B, Engebretsen IMS (2021) Iron deficiency anemia among children aged 2–5 years in southern Ethiopia: a community-based cross-sectional study. PeerJ9: e11649. doi: 10.7717/peerj.11649 34249504 PMC8247708

[49] Orsango AZ, Loha E, Lindtjørn B, Engebretsen IMS (2021) Co-morbid anaemia and stunting among children 2–5 years old in southern Ethiopia: a community-based cross-sectional study. BMJ Paediatrics Open5: e001039. 10.1136/bmjpo-2021-001039. PMC8215259 34222679

[50] Gosdin L, Martorell R, Bartolini RM, Mehta R, Srikantiah S, et al. (2018) *The co-occurrence of anaemia and stunting in young children*. 14: e12597. 10.1111/mcn.12597. PMC6866136 29468825

[51] Tessema M, Belachew T, Ersino G (2013), Feeding patterns and stunting during early childhood in rural communities of Sidama, South Ethiopia. Pan African Medical Journal14. doi: 10.11604/pamj.2013.14.75.1630 23646211 PMC3641921

[52] Desalegn BB, Jagiso B (2020) Low diet diversity and its associated factors among the mothers and their children in agroforestry land use systems of Sidama, Ethiopia: A community-based cross-sectional study. Cogent Food & Agriculture6: 1818367. 10.1080/23311932.2020.1818367.

[53] Berra WG (2020) Household Food Insecurity Predicts Childhood Undernutrition: A Cross-Sectional Study in West Oromia (Ethiopia). J Environ Public Health, 2020: 5871980. doi: 10.1155/2020/5871980 32211049 PMC7085371

[54] Gibson RS, Abebe Y, Hambidge KM, Arbide I, Teshome A, et al. (2009) Inadequate feeding practices and impaired growth among children from subsistence farming households in Sidama, Southern Ethiopia. Matern Child Nutr5: 260–75. 10.1111/j.1740-8709.2008.00179.x.djk PMC6860599 20572929

