# Supplementary material for: Dietary practices and nutritional status of young children in the former ensete monoculture dominated Sidama region, southern Ethiopia: A community based cross-sectional study
Source: PLoS One. 2022 Sep 14;17(9):e0272618. doi: 10.1371/journal.pone.0272618 (PMC9473397; doi:10.1371/journal.pone.0272618)
Supplement: S1 Table — (PDF) [file pone.0272618.s001.pdf]

S1 Table. Descriptive findings of continuous variables, Dale district, Ethiopia, 2018.

Continuous variables	N	Mean	SD	Minimum	Maximum
Child age in months	903	14.1	6.3	0.8	23.9
Birth order	903	2.6	1.6	1	9
LAZ	903	-1.6	1.7	-6.2	3.5
WLZ	903	0.7	1.6	-5.2	4.9
Child hemoglobin	901	10.6	1.4	5.5	14.2
Dietary diversity score	742	4.3	1.9	1	9
Age of the mother	897	26.9	5.1	16	45
Height of the mother	897	157.9	7.0	136	177.4
Mother's hemoglobin	897	13.5	1.3	7.9	17
HFI score	903	4	3.2	0	19
Household size	903	4.8	1.6	3	11

IQR=interquartile range, LAZ=length-for-age z-score, WLZ=weight-for-length z-score, and HFI= household food insecurity
