# Supplementary material for: Dietary practices and nutritional status of young children in the former ensete monoculture dominated Sidama region, southern Ethiopia: A community based cross-sectional study
Source: PLoS One. 2022 Sep 14;17(9):e0272618. doi: 10.1371/journal.pone.0272618 (PMC9473397; doi:10.1371/journal.pone.0272618)
Supplement: S2 File — (PDF) [file pone.0272618.s003.pdf]

Questionnaire (English version)

Questionnaire prepared to gather selected socio-demographic, nutrition, and health status information about children aged below 24 months, mothers aged 15- 49 years. The questionnaire contains three sections and directions in each part.

Question identification number _____________

Section one

Questions about general condition of the household

0. House identification

001	Date / / (day/ month/ year)
002	Kebele
003	Village/subkebele
004	House identification number
005	Question identification number
006	Data collector identification number
007	Supervisor identification number

1. Household background

101	How many people live in this house? (identify age and sex)	Year	Male	Female	
		< 5 years			
		5-18 years			
		18-65 years			
		65 years and above			
		Total			
103	Who is the head of the household?	1. The father and the mother<br>2. The mother only<br>3. The father only<br>4. Other/write_________________			
104	Choose from the given properties available in your house (You can choose more than one)	1.Television ________<br>2.Refrigator _________<br>3.Mobile phones _______<br>4. Motor bike _______<br>5. Bicycle ________<br>6. Car ______<br>7. Nothing available ________			
105	Choose from the given properties available in your house (You can choose more than one)	1. Hens _____<br>2. Goat _____<br>3. Sheep _____<br>4. Cows _____<br>5. Bulls/Ox _____<br>6. Donkey _____<br>7. Horse _____			

2. Feeding habit of the family

201	What is the common food used by the family?	1. Ensete/ ‘Kocho’______<br>2. Maize/corn _____<br>3. Potato, Sweet potato, ‘Boina’ and other root foods _____<br>4. Other/ write ______		
202	Is there any food taboo in the family?	1. No _____<br>2. Yes _____		
203	If your answer is yes, describe the type of food and reason for the taboo?	Type of food	Reasons for the taboo	
204	Is there any food taboo to children?	1. No _____<br>2. Yes _____		
205	If your answer is yes, describe the type of food and reason for the Prohibition?	Type of food	Reason for the prohibition	
206	Is there any food taboo to mothers, pregnant and breast feeding mothers?	1. No _____<br>2. Yes _____		
207	If your answer is yes, describe the type of food and reason for the Prohibition?	Type of food	Reason for the prohibition	
208	What is common water source of the family?	1. Pipe water<br>2. Open pond<br>3. Closed pond<br>4. Running river<br>5. Other/ write ____________		

3. Household’s food insecurity access scale (HFIAS) measurement

301	Do you trouble because there is no enough food to the family in the past one month?	0. No, I don’t<br>1. Yes, I do	If your answer is no, go to Q.N. 303
302	How long the problem sustained in the past one month?	1.Occasionally (one or two times) ___<br>2. Sometimes (three to four times) ___<br>3. Many times (above ten times) ___	
303	Do you or one of your family members missed a meal because of absence of food even if you want to eat in the past one month?	0. No, I don’t<br>1. Yes, I do	If your answer is no, go to Q.N. 305
304	How long the problem sustained in the past one month?	1.Occasionally (one or two times) ___<br>2. Sometimes (three to four times) ___<br>3. Many times (above ten times) ___	
305	Do you or one of your family members eat the same type of meal repeatedly because of absence of food even if you want to eat in the past one month?	0. No, I don’t<br>1. Yes, I do	If your answer is no, go to Q.N. 307
306	How long the problem sustained in the past one month?	1.Occasionally (one or two times) ___<br>2. Sometimes (three to four times) ___<br>3. Many times (above ten times) ___	
307	Do you or one of your family members eat a meal you never want to eat because of absence of food in the past one month?	0. No, I don’t<br>1. Yes, I do	If your answer is no, go to Q.N. 309
308	How long the problem sustained in the past one month?	1.Occasionally (one or two times) ___<br>2. Sometimes (three to four times) ___<br>3. Many times (above ten times) ___	
309	Do you or one of your family members eat small amount of meal even if you want more because of shortage of food in the past one month?	0. No, I don’t<br>1. Yes, I do	If your answer is no, go to Q.N. 311
3010	How long the problem sustained in the past one month?	1.Occasionally (one or two times) ___<br>2. Sometimes (three to four times) ___<br>3. Many times (above ten times) ___	
3011	Do you or one of your family members eat small amount of meal within a day because of shortage of food in the past one month?	0. No, I don’t<br>1. Yes, I do	If your answer is no, go to Q.N. 313
3012	How long the problem sustained in the past one month?	1.Occasionally (one or two times) ___<br>2. Sometimes (three to four times) ___<br>3. Many times (above ten times) ___	
3013	Do you experience total absence of food in the house because of deprivation in the past one month?	0. No, I don’t<br>1. Yes, I do	If your answer is no, go to Q.N.315

3014	How many times the problem occurs in the past one month?	1.Occasionally (one or two times) ___<br>2. Sometimes (three to four times) ___<br>3. Many times (above ten times) ___	
3015	Do you one of your family members missed diner because of absence of food in the house in the past one month?	0. No, I don't<br>1. Yes, I do	If your answer is no, go to Q.N. 317
3016	How many times the problem occurs in the past one month?	1.Occasionally (one or two times) ___<br>2. Sometimes (three to four times) ___<br>3. Many times (above ten times) ___	
3017	Do you one of your family members missed a meal the whole day in the past one month?	0. No, I don't<br>1. Yes, I do	If your answer is no, go to Q.N. 319
3018	How many times the problem occurs in the past one month?	1.Occasionally (one or two times) ___<br>2. Sometimes (three to four times) ___<br>3. Many times (above ten times) ___	
3019	Yesterday, was there unusual meal in your house or neighbor because it was a special day	1. Yes<br>2. No	
3020	What is the main food source of the family?	1. Private farming, fishing<br>2. Buying food<br>3. Job for food, support from relatives<br>4. Food support from organizations<br>5. Other/ describe ___________	

Section two

Questions referring children less than 24 months of age

4. General information and child feeding practice

S.N	Question	Response	
401	Sex of the child	Male _____<br>Female _____	
402	The relationship of the respondent with the child	1. Biological mother ____<br>2. Biological father ____<br>3. Step mother ____<br>4. Step father _____<br>5. Other (specify) _______________	
403	Age of the child	_____ Months	
404	Date of birth	___/___/_____ (DD/MM/YYYY)	
405	Date of birth is confirmed with vaccination card or birth certificate	1. Yes ____<br>2. No ____	
406	Birth order of the child	_______________	
407	Place of birth	1. Home _____<br>2. Health institution (specify) ________________________<br>3. Other (specify) ______________	
408	Birth weight;	____________ kg	

409	Birth weight confirmed with vaccination card or birth certificate	1. Yes _____<br>2. No ______	
4010	Is your child currently breast feeding?	1. Yes _____<br>2. No _____	
4011	If no, what is the reason	1. Mother's health related _____<br>2. Mother's job related_____<br>3. Child health related_____<br>4. Satisfied with breast milk (age) ____<br>5. Separated from mother ____<br>6. Current pregnancy ____<br>7. Other (specify) ___________________	
4012	When did you start breast feeding to the child	1. Immediately after birth _____<br>2. Within one hour after birth ____<br>3. After one hour from birth _____<br>4. After 24-hour from birth _____<br>5. Other(specify) ______________	
4013	Did you give any Pre-lacteal feed to the child?	1. Yes _____<br>2. No _____	If no, go to Q416
4014	If yes, what was given?	1. Formula milk _________<br>2. Cow's milk ____________<br>3. Sugar and water_________<br>4. Other(specify) _________________	
4015	Did you use bottle feeding to your child?	1. Yes _____<br>2. No _____	
4016	Is this child stated complementary feeding	1. Yes ______<br>2. No ______	If no, go to Q419
4017	If yes, at what age the child started complementary feeding?	At ______________months	

5. The dietary diversity of the child

'Did this child ate (name of food) yesterday during the day and night?			
501	Bread, injera, porridge, or other foods made from cereals like;<br>Maize, bread, sorghum, teff, wheat, barley, rice, noodles/indomi,	1. No _____<br>2. Yes _____	
502	Commercial fortified infant or baby food (e.g. Cerelac, Fafa)?	1. No _____<br>2. Yes _____	
503	Pumpkin, carrots, squash, or sweet potatoes that are yellow or orange inside?	1. No _____<br>2. Yes _____	
504	White potatoes, sweet potato, kocho/bulla, white		

	yams, cassava, or other foods made from roots/tubers?	1. No _____<br>2. Yes _____	
505	Any dark green leafy vegetables (e.g. kale)	1. No _____<br>2. Yes _____	
506	Any other vegetables?	1. No _____<br>2. Yes _____	
507	Ripe mangoes, papayas, or other orange colored fruits?	1. No _____<br>2. Yes _____	
508	Any other fruits?	1. No _____<br>2. Yes _____	
509	Liver, kidney, heart, or other organ meat?	1. No _____<br>2. Yes _____	
5010	Any meat, such as beef, pork, lamb, goat, chicken?	1. No _____<br>2. Yes _____	
5011	Eggs?	1. No _____<br>2. Yes _____	
5012	Fish?	1. No _____<br>2. Yes _____	
5013	Any foods made from beans, peas, lentils, or nuts?	1. No _____<br>2. Yes _____	
5014	Cheese or other food made from milk (e.g. yogurt)?	1. No _____<br>2. Yes _____	
5015	Other fats or oils (e.g., vegetable oil, ghee, 'shenokibe') or foods made with them?	1. No _____<br>2. Yes _____	
5016	Sugar and other sugary products (e.g. chocolates, biscuit, sweets, candies, soda)	1. No _____<br>2. Yes _____	
5017	Any other semi-solid, solid, or soft/mashed foods that I have not mentioned?	1. Write the food _________________	

6. Anthropometric measurements and hemoglobin level of the child

601	Weight (in kg)	________________ Kg	
602	Height/length (in cm)	_________________ Cm	
603	Hemoglobin level	_________________ g/dl	

Section Three

Questions about the mother aged 15-49 years

7. Reproductive characteristics and general information of the index mother

701	Age of the mother	_______ years	
702	What is highest completed level of your education?	1. No schooling at all _____<br>2. Completed Grade _______<br>3. Certificate/ diploma level _____<br>4. Degree and above____	
703	Are you employed	Yes ______ No _______	
704	Total number of pregnancy	___________	
705	Age at first pregnancy	___________ years old	
706	Currently pregnant?	1. Yes ______<br>2. No ______<br>3. I don't know ______	
707	Hemoglobin level of the mother	__________ g/dl	
