# Supplementary material for: Dietary practices and nutritional status of young children in the former ensete monoculture dominated Sidama region, southern Ethiopia: A community based cross-sectional study
Source: PLoS One. 2022 Sep 14;17(9):e0272618. doi: 10.1371/journal.pone.0272618 (PMC9473397; doi:10.1371/journal.pone.0272618)
Supplement: S3 File — (PDF) [file pone.0272618.s004.pdf]

Questionnaire (Sidaamu Afoo version)

Tini xa’mo dirinsa 24 agani woroo no qaaqquliranna dirinsa 15 - 49 mereero no amuwi itate gara kikkisate kaa’litanno taje xinqate qixeesinoonte. Xa’mubba aantinotta sase kifile amaddinoha ikkana xa’mo xa’matenna taje borreesate injaanno gede mitte mittente kifilera massago uyinoonni

Xa’mubbate badooshshi ______________

Umikki kifile

Maatete xaphphoomu gara la’ano xa’mubba

0. Minu badooshshe

001	Barra ___/___/____(barra/agana/diro)
002	Ollaa ________________
003	Nafara _______________
004	Minu badooshshi kiiro ______
005	Xa’mote badooshshi kiiro:_______
006	Taje xinqannohu badooshshi kirro:__________
007	Towaatannohu kiiro:________

1. Dagoomunna miinju xa’mubba

101	Konne mine xaphphoomunni meu manni hee’ranno/dirunninna koo/ Teenni bade borreessi/	Diro	Labaaha	Meyaata	
		5 diri woro			
		5-18 diri geeshsha			
		18-65 diri geeshsha			
		65 diri ale			
		Xaphphooma			
103	Kuni mini magarinni massagamanno?	1. Amanna annu massagganno<br>2. Ama callichose massaggannoho<br>3. Annu callichchisi massagannoho<br>4. 4. wolu nooro kuli_______________			
104	Uyiinoonni doorshi giddonni, minekki nooha kuli (mittu ale doora dandiinanni)	1. Televizhine _____<br>2. Firiije_____<br>3. Milli yitanno bilbilla_____<br>4. Moteri biskiliite_____<br>5. Biskiliite _____<br>6. Kameelu_____			

		7. Noohu dino_____	
105	Uyiinoonni doorshi giddonni, minekki nooha kuli/mittu ale doora dandiinanni/	1. Lukkuwa _____<br>2. Me’’e _____<br>3. Gerecho _____<br>4. Adote saa _____<br>5. Booto woyi hando _____<br>6. Haricho _____<br>7. Farasho _____	

2. Xaphphoomu maatete itate gara

<table>
<tr><td>201</td><td>Maatete duuchchu woyiti sagale maati</td><td colspan="2">1. Waasa _____
2. Badala _____
3. Dinnichcha, baxaaxeessa, bohenna wole rumudaame sagale _____
4. Wolu hee’riro kuli ________________</td><td></td></tr>
<tr><td>202</td><td>Maatete hoollanni sagale no?</td><td colspan="2">1. Dino _____
2. Ee no _____</td><td></td></tr>
<tr><td rowspan="4">203</td><td rowspan="4">Dawaro ee ikkituro hoollanni sagalenna korkaata kuli</td><td>Sagalete dana</td><td>Hooloonni korkaata</td><td rowspan="4"></td></tr>
<tr><td></td><td></td></tr>
<tr><td></td><td></td></tr>
<tr><td></td><td></td></tr>
<tr><td>204</td><td>Qaaqqulleho hoollanni sagale no?</td><td colspan="2">1. Dino _____
2. Ee no _____</td><td></td></tr>
<tr><td rowspan="4">205</td><td rowspan="4">Dawarokki ee ikkituro, hoollanni sagalenna hoollanni korkaata kuli</td><td>Sagalete dana</td><td>Hooloonni korkaata</td><td rowspan="4"></td></tr>
<tr><td></td><td></td></tr>
<tr><td></td><td></td></tr>
<tr><td></td><td></td></tr>
<tr><td>206</td><td>Mine amuwaho, godowinni nooriranna qansi’rano amuwira hoollanni sagale no</td><td colspan="2">1. Dino _____

2. Ee no _____</td><td></td></tr>
<tr><td rowspan="5">207</td><td rowspan="5">Dawaro ee ikkituro hooloonni sagalenna hooloonni korkaata kuli</td><td>Sagalete dana</td><td>Hooloonni korkaata</td><td rowspan="5"></td></tr>
<tr><td></td><td></td></tr>
<tr><td></td><td></td></tr>
<tr><td></td><td></td></tr>
<tr><td></td><td></td></tr>
<tr><td>208</td><td>Maatete wayii buichcho mamaati?</td><td colspan="2">1. Baanba
2. Fano balenni
3. Diwantino balenni</td><td></td></tr>
</table>

		4. Ha'ranno wayiinni, buichchotenni<br>5. Wolu hee'riro kuli _____________	

3. **Sa'u shoolle lamala giddo sagaleteni umo dandaa laino xa'mo**

301	Sai mittu again giddo maatete ikkitanno sagale dino yite qarrante egenootta?	0. Diegenoomma<br>1. Ee	Dawaro diegenoomma ikkituro, X. K. 303 sai
302	Sai agani giddo kuni gari mageeshshi yannara iilli?	1. Sae sae (mitte woyi lame hige) ___<br>2. Mitte mitte hige (sauyi shoolu geeshsha) ___<br>3. Duuchcha hige (Tonnu ale) ___	
303	Sai again giddo ati woyi maatekkinni mittu hoongunni kainohunni sagale itate hasidhine ittinikki gattine egentinoonni?	0. Diegenninoommo<br>1. Ee	Dawarokki diegeninoomm o ikkituro, X. K. 305 sai
304	Sai again giddo kunirichchi mageeshshi yannara kalaqami?	1. Sae sae (mitte woyi lame hige) ___<br>2. Mito mito woyiite (3 - 4 hige) ___<br>3. Duuchcha hige (tonnu ale) ___	
305	Sai mittu again giddo ati woyi maatekki mereero hoongunni kainohunni gama sagale calla qoltine qoltine ittine egentinoonni?	0. Diegenninoommo<br>1. Egenninoommo	Dawarokki diegeninoomm o ikkiro X. K. 307 sai
306	Sai aganira kunirichchi mageeshshi yannara ikki?	1. Sae sae (mitte woyi lame hige) ___<br>2. Mito mito woyite (3 – 4 hige) ___<br>3. Duuchcha hige (tonnu ale) ___	
307	Sai mittu again giddo ati woyi maatekkinni mittu wole sagale afi'ra dandiitinoonnikkihura horonta itate hasidhinannikki sagale ittine egentinoonni?	0. Diegeninoommo<br>1. Egeninoommo	Dawarokki diegeninoomm o ikkituro X. K. 309 sai
308	Sai again giddo kunirichchi mageeshshi yannara ikki?	1. Sae sae (mitte woyi lame hige) ___<br>2. Mitto mitto woyiite (3 – 4 hige) ___	

		3. Duuchcha hige (tonnu ale) ___	
309	Sai mittu agani giddo ati woyi maatekkinni mittu ikkitanno sagale nookkihura barrunni ita noonke yitine heddinannihuni ajjino sagale ittine egentinoonni?	0. Diegeninoommo<br>1. Egeninoommo	Dawarokki diegeninoommo ikkituro, X. K. 311 sai
310	Sai again giddo kunirichchi mageeshshi yannara ikki?	1. Sae sae (mitte woyi lame hige) ___<br>2. Mito mito woyite (3 – 4 hige) ___<br>3. Duuchcha hige (tonnu ale) ___	
311	Sai mittu again giddo ati woyi maatekkinni mittu mine ikkitanno sagale hoogatenni barra wo'ma shiima sagale calla ittine egentinoonni?	1. Diegeninoommo<br>2. Egeninoommo	Dawarokki diegeninoommo ikkiro, X. K. 313 sai
312	Sai again giddo kunirichchi mageeshshi yannara ikki?	1. Sae sae (mitte woyi lame hige) ___<br>2. Mito mito woyiite (3 – 4 hige) ___<br>3. Duuchcha hige (tonnu ale) ___	
313	Sai mittu agani giddo hoongunni kainohunni minekki mitturino intanni sagale ba'e egentino?	1. Diegentino<br>2. Egentino	Dawarokki diegentino ikkituro, X. K. 315 sai
314	Sai again giddo kunirichchi mageeshshi yannara ikki?	1. Sae sae (mitte woyi lame hige) ___<br>2. Mito mito woyite (3 – 4 hige) ___<br>3. Duuchcha hige (tonnu ale) ___	
315	Sai mittu again giddo ati woyi maatekkinni mittu ikkitanni sagale hoogatenni hurbaate ittinikki goxxine egentinoonni?	1. Diegeninoomme<br>2. Egeninoommo	Dawarokki diegeninoommo ikkituro, X. K. 317 sai
316	Sai agani giddo kunirichchi mageeshshi yannara ikkI?	1. Sae sae (mitte woyi lame hige) ___<br>2. Mito mito woyite (3 – 4 hige) ___<br>3. Duuchcha hige (Tonnu ale) ___	
317	Sai mittu again giddo ati woyi maatekkinni mittu barra wo'ma sagale horo ittinikki gattine egentinoonni?	1. Diegenninoommo<br>2. Egeninoommo	Dawaro diegenninoommo ikkituro, X. K. 319 sai
318	Sai agani giddo kunirichchi mageeshshi yannara ikki?	1. Sae sae (mitte woyi lame hige) ___<br>2. Mito mito woyite (3 – 4 hige) ___	

		3. Duuchcha hige (tonnu ale) ___	
319	Be’ro barra minekki woyi ollaaho ayyaana woyi baxxino barra ikkinohura albiwi baxxino itate gari no?	1. Ee<br>8. Dino	
320	Maatete waanna sagalete buichcho maati?	1. Gillete gatinni gamba assate, qulxu’me woshshaadate<br>2. Hidhate<br>3. Sagale loosoho loosate, fiixa kaa’lo xa’mirate<br>4. Sagale kaa’litanno diriijitenni<br>5. Wolu hee’riro kuli___________	

Layiinkki kifile

Xiinxallote eate dooramannoha dirisi 24 agani woroonni ikkino qaaqqo la’ano xa’mubba

4. Qaaqqu xaphphoomu tajenna itate gara

401	Qaaqqu koo/tee	Labbaaho________<br>Meyaate________	
402	Xa’minanni manchi qaaqqu ledo noosi fiixoomi gara	1. Iltinno ama<br>2. Ilinno anna<br>3. Buddeenu ama<br>4. Buddeenu anna<br>5. Wolu hee’riro kuli___________	
403	Diro	________ aganna	
404	Qaaqqu ilamino barra	___/___/______(barra/agana/diro)	
405	Qaaqqu ilamino barri kittibaatete/ilamate kaardenni buuxamino	1. Ee ______<br>2. Dibuuxamino ______	
406	Kuni qaaqqi meikkiho;	______kki qaaqqooti.	
407	Kuni qaaqqi mama ilamino	1. Mini giddo ___<br>2. Fayyimate uurrinshara (xawisi) ____________<br>3. Wolu hee’riro kuli ___________	

408	Qaaqqu ilami woyite mageeshsha ayiirranno	________ kiilo giraame	
409	Qaaqqu ilami woyiite noo ayiirra kibaatete/ilamate kaardenni buunxoonni	3. Ee______<br>4. Dibuunxoonni ______	
4010	Qaaqqu unuuna qananno	1. Ee qananno ______<br>2. Diqananno ______	
4011	Dawarokki diqananno ikkituro, korkaata kuli	1. Amate faayyima ledo amadaminoha<br>2. Amate loosi ledo Amadaaminoha<br>3. Qaaqu faayyimaa ledo Amadaaminoha.<br>4. Qaaqu Amate unuunin duuwa<br>5. Amatewin baxiro<br>6. xa godowin hedhuuro<br>7. Wolu heriro kuli	
4012	Qaaqqu ilamihunni mageeshshi yanna gedensaanni qani	1. Ilamanni hee're<br>2. Ilamihunni mitte sa"ate giddo<br>3. Ilamihunni mitte sa"ate gedensaanni<br>4. Ilamihunni lemiina shoole sa"ate gedensaanni<br>5. Wolu hee'riro kuli __________	
4013	Qaaqqu ilamanni hee'reenna unuunu gobaanni woluri uyinoonnisiri no?	1. Ee<br>2. Uyiinoonnisiri dino	
4014	Dawarokki ee ikkituro, uyiinoonnisiri maatiro kuli	1. Bulleete ado ______<br>2. Saadate ado______<br>3. Waanna sukaare______<br>4. Wolu hee'riro kuli __________	
4015	Qaaqqu xuunxo qanino/qane egennino?	1. Ee______<br>2. Qane diegenino______	
4016	Qaaqqu ledote sagale adha hanafino?	1. Ee hanafino______<br>2. Dihanafino______	
4017	If yes, at what age the child started complementary feeding?	At ______________months	

5. Qaaqqu itate taalle lainohunni

**Qaaqqu aantete kuloonniri giddo bero barra, hawarronna hashsha hiikkuri itino?**			
501	Badalate, bashanqunni, gaashshete, sindete, hayiixunni loonsoonni huuffisa woyi sherko, budeena, daabbo, ruuze	1. Ee itino______<br>2. Di”itino______	
502	Xaanxe hirranni sagale (lawishshaho, faaffa, serelake)	1. Ee itino______<br>2. Di”itino_____	
503	Baaqula, karoote, baxaaxeessa woyi danansa duumo woyi haanja ataakilte	1. Ee itino______<br>2. Di”itino______	
504	Dinnicha, baxaaxeessa, waasa, bulla, bohe, kasaava, woyi wole rumudaame sagale	1. Ee itino______<br>2. Di”itino_____	
505	Daninsa kolishirino ataakilte lawishsha ho hawashu shaana	1. Ee itino______<br>2. Di”itino______	
506	Wolu ittinohu atikiltete sirchi no?	1. Ee itino______<br>2. Di”itino______	
507	Maango, papaayya woyi wole baqqala dana afidhino gumma	1. Ee itino______<br>2. Di”itino______	
508	Wole gumma lawishshaho awukaato	1. Ee itino______<br>2. Di”itino______	
509	Afale, mule, wodana	1. Ee itino______<br>2. Di”itino_____	
5010	Maala, handunniha, lukkichcho, gerechcho, meichcho, manchaame	1. Ee itino______<br>2. Di”itino______	
5011	Quuphphe	1. Ee itino______<br>2. Di”itino______	
5012	Qulxu’me	1. Ee itino______<br>2. Di”itino______	
5013	Baaqeelunni, shunburunni, atarunni, missirunni, ochcholonete loonsoonni sagale	1. Ee itino______<br>2. Di:itino______	
5014	Adoteni, geintuni, Ayiibeteni/burbuxxo?	1. Ee itino______<br>2. Di:itino______	
5015	Zayitete woyi buurunni loonsoonni sagale	1. Ee itino______<br>2. Di”itino______	
5016	Coomaworichco lawishshaho chokkolete, biskuute, karameella, shaqado ago mirinda, peepisi, koolla	1. Ee itino______<br>2. Di”itino______	
5017	Wolu qaaqqu itinohunna kulloonnikkihu hee’riro kuli	1. __________<br>2. __________<br>3. __________	
5018	Be’ro barra qaaqqu me”e hige iti?	________(kiirote borreessi)	

6. Qaaqqu mannimate bikkanna mundeete anje buuxo guma

601	Ayiirra kilo giraamete	__________ K. G.
602	Hojja/seendille S. M	__________ S. M
603	Heemogilobinete deerra	__________ g/dl

Sayikki kifile

Xiinxallote eate doorantinori dirinsa15-49 ikkino meento la'ano xa'mubba

7. Xaphphomu tajena ilate gara lainohunni

	Uyinoonni doorshi giddo ate dawaro albaanni malaate assi woyi uyinoonnihe darga dawaro wonshi.		
701	Dirikki me"eho	________ diro	
702	Gudoottati jawiidi rosi deerra	1. Horo rosse egentinkkiti _____<br>2. kifile deerra guddinoti _____<br>3. Sertifikeetete/dipiloomunni maassantino _____<br>4. Digirenna hakuyi ali rosu deerra _____	
703	Damoozunni qaxaramoottoho	Dee'ni _____<br>Ee _____	
704	Me"e higge godowootta?	__________	
705	Umo godowitta waro dirikki me"eho?	_________ diro	
706	Xa buuxamino godowi no?	1. Ee _____<br>2. Dino _____<br>3. Diafoomma ________	
707	Heemogilobinete deerra	__________ g/dl	
